# Acute Macular Neuroretinopathy in Purtscher Retinopathy

**DOI:** 10.4274/tjo.galenos.2019.02488

**Published:** 2020-04-29

**Authors:** Berrak Şekeryapan Gediz

**Affiliations:** 1 University of Health Sciences, Ankara Ulucanlar Eye Training and Research Hospital, Clinic of Ophthalmology, Ankara, Turkey

**Keywords:** Purtscher retinopathy, acute macular neuroretinopathy, optical coherence tomography, optical coherence tomography angiography, ischemia

## Abstract

Purtscher retinopathy and acute macular neuroretinopathy are two rare clinical disorders that are both probably associated with ischemic pathogenesis. In this report, we describe for the first time the coexistence of Purtscher retinopathy and acute macular neuroretinopathy in a patient with visual complaints after chest trauma. Optical coherence tomography (OCT) scans demonstrated outer retinal defects, while OCT angiography illustrated areas of hypoperfusion in the superficial and deep capillary plexuses as well as the choriocapillaris. In this report, it is emphasized that acute macular neuroretinopathy is a clinical condition that should be kept in mind in patients presenting with post-traumatic vision loss. Although its clinical diagnosis is difficult, characteristic OCT and OCT angiography findings facilitate diagnosis.

## Introduction

Purtscher retinopathy is a rare condition characterized by vision loss and central visual field defects that develops after traumas that do not involve direct contact with the eyes, such as chest and head trauma. Clinical findings include peripapillary cotton-wool spots, areas of retinal whitening (Purtscher flecken), and hemorrhages. Although the pathogenesis remains unclear, the findings are believed to arise due to retinal ischemia caused by embolic occlusion of the precapillary arterioles.^1,2^

Acute macular neuroretinopathy (AMN) is an uncommon clinical entity characterized by dark intraretinal lesions accompanied by acute-onset paracentral scotomas. It was first described in young women using oral contraceptives, and later publications demonstrated it could also be associated with viral infections, trauma, surgery, or medications.^3,4,5^ Retinal microvascular changes are implicated in the pathogenesis of AMN.^6^ Optical coherence tomography (OCT) and OCT angiography (OCTA) findings reveal the presence of superficial and/or deep capillary plexus and choriocapillaris ischemia in AMN.^6,7,8,9,10^

Although there have been a few previous reports in the literature of AMN associated with trauma, here we present for the first time a patient who developed AMN due to Purtscher retinopathy, with findings of nonperfusion in both the superficial and deep capillary plexuses and choriocapillaris.

## Case Report

**Figure 1:**
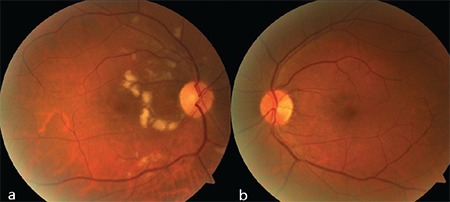
**a)** Color fundus photograph of the right eye shows cotton-wool spots in the peripapillary region, intraretinal hemorrhages, and a dark lesion with indistinct borders located in the central fovea. **b)** Color fundus photograph of the left eye shows a dark lesion located in the nasal fovea that does not cross the vertical midline

**Figure 2:**
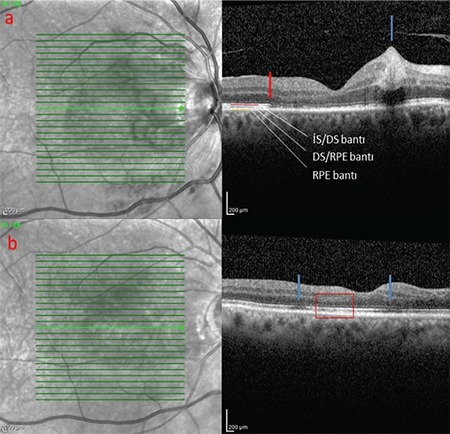
**a)** Infrared image of the right eye and OCT section through the lesion. The outer retinal layers are individually marked. The point where loss of the IS/ OS and OS/RPE bands begins is indicated with a red arrow; the hyperreflective thickening caused by soft exudate is shown with a blue arrow. **b)** In the OCT image of the same eye 6 months later, areas of OS/RPE band loss are indicated with blue arrows. The IS/OS band is faint in these areas. The area of outer nuclear layer thinning is indicated with a rectangle OCT: Optical coherence tomography

**Figure 3:**
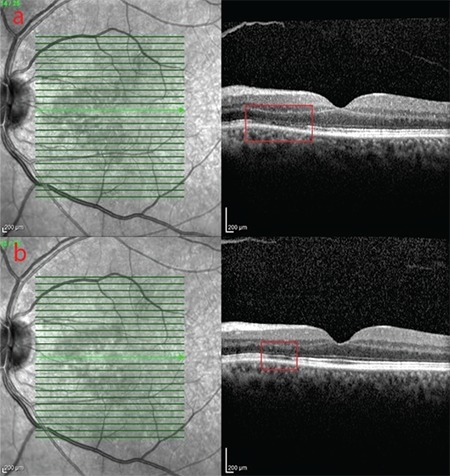
**a)** Infrared image of the left eye and OCT section through the lesion. In the section through the hyporeflective lesion in the nasal fovea, loss of the IS/ OS and OS/RPE bands is marked with a rectangle. **b)** OCT image of the same eye 6 months later shows the indicated area decreased in size and the IS/OS band reappeared, but loss of the OS/RPE band persists OCT: Optical coherence tomography

**Figure 4:**
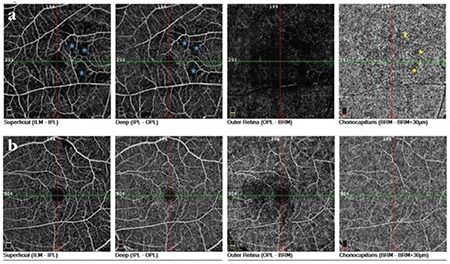
**a)** OCTA image of the right eye. Areas marked with a blue star indicate superficial and deep capillary plexus hypoperfusion; areas marked with a yellow star indicate choriocapillaris hypoperfusion corresponding to the areas of retinal hypoperfusion. **b)** OCTA image of the left eye appears normal OCTA: OCT angiography

A 61-year-old man presented with complaints of decreased vision in the right eye and central scotoma in the left eye. He reported having an intravehicular traffic accident 15 days earlier and that his visual complaints started immediately after the accident. General physical examination was unremarkable except for a fractured rib. On ophthalmologic examination, his best corrected visual acuity (BCVA) was 20/400 in the right eye and 20/20 in the left eye. Intraocular pressures and anterior segment findings were normal in both eyes. Fundus examination revealed cotton-wool spots in the peripapillary region, intraretinal hemorrhages, and dark lesions with indistinct borders in the central fovea of the right eye. On examination of the left eye, it was seen that the same dark lesion was present in the nasal fovea and did not cross the vertical midline (Figures 1a and b). An infrared image of the right eye revealed a hyporeflective foveal lesion; the OCT section passing through the lesion showed hyperreflective thickening of the ganglion cell and nerve fiber layers caused by soft exudates, in addition to loss of the subfoveal photoreceptor inner segment/outer segment (IS/OS) band and photoreceptor outer segment/retinal pigment epithelium (OS/RPE) band (Figure 2a). On the infrared image of the left eye, the borders of a hyporeflective lesion located in the nasal fovea were clearly visible, while the OCT section corresponding to the lesion revealed losses in the IS/OS and OS/RPE bands (Figure 3a). The patient was followed for a diagnosis of AMN secondary to Purtscher retinopathy. At 6-month follow-up, his BCVA was 20/40 in the right eye and 20/20 in the left eye, and the central scotoma in his left eye had disappeared. The infrared image of the right eye demonstrated that the borders of the lesion had shrunk, while OCT showed that the IS/OS band was visible although still faint in places, the OS/RPE band was visible except in two localized areas, and the outer nuclear layer had thinned (Figure 2b). Similarly, the infrared image of the left eye showed that the borders of the lesion had shrunk, while OCT revealed that the IS/OS band had reappeared and the defect in the OS/RPE band had diminished in size (Figure 3b). OCTA sections revealed superficial and deep capillary plexus hypoperfusion and reduced flow in the choriocapillaris corresponding to the areas of retinal hypoperfusion in the right eye (Figure 4a). Evaluation of the left eye was normal (Figure 4b).

## Discussion

Purtscher retinopathy is believed to develop as a result of leukocyte aggregation due to trauma-related activation of the complement system. These aggregates cause occlusion and secondary infarction in the retinal arterioles. Occlusion of the precapillary arterioles results in cotton-wool spots when it involves the superficial capillary network and Purtscher flecken when the deep capillary network is affected.^1,2^

AMN is a rare disease first described in 1975 by Bos and Deutman^3^ based on clinical observation and fundus fluorescein angiography findings. The acute onset of the disease and the presence of risk factors such as infection, inflammation, and ischemia suggested a vascular etiology. With the development of multimodal imaging techniques, the pathogenesis of AMN has become clearer and OCT started to be used in diagnosis.^4,5,6^ The OCT findings of AMN can be separated into the early and late period. Early findings include the appearance of a hyperreflective band in the outer plexiform and outer nuclear layer, disruption of the IS/OS and OS/RPE lines, and the hyperreflective band usually regresses within 1 week. In the late period, the IS/OS line reappears, OS/RPE disruption continues, and permanent thinning of the outer nuclear layer is added to the findings. In light of OCT findings, deep capillary plexus ischemia is believed to be responsible for the pathogenesis.^6^ Our patient presented all OCT findings other than the hyperreflective band in the outer nuclear layer that appears and disappears in the early period. This hyperreflective band may not have been observed because the patient presented 2 weeks after the trauma.

With the introduction of OCTA, the hypoperfusion involved in the pathogenesis of AMN has been more clearly demonstrated. However, the vascular region in which hypoperfusion is observed varies in different publications. While some publications report that choriocapillaris ischemia alone is responsible for the pathogenesis, there are also cases in which superficial and/or deep retinal perfusion is affected with no impact on the internal choroid layers.^7,8,9,10^ OCT findings of outer nuclear layer thinning subsequent to early changes in the outer plexiform layer indicate that hypoperfusion in the choriocapillaris as well as the deep capillary plexus is responsible for the pathogenesis. This suggests that the hypoperfusion in AMN may occur at the level of the ophthalmic artery, thus affecting both the deep capillary plexus and choriocapillaris.^11^ Both superficial and deep capillary plexus and choriocapillaris hypoperfusion was observed in our patient’s right eye. The appearance of cotton-wool spots demonstrates that ischemia also affects the superficial capillary plexus.

A few cases of AMN developing after trauma have been reported in the literature. The association between indirect trauma and AMN was first demonstrated clinically by Gillies et al.^12^, who reported that the increase in intrathoracic pressure resulting from an accident caused an elevation in intravascular pressure, thereby resulting in acute disruption of the blood-retina barrier. Later case series presented the OCT findings of AMN and reported that hypotension or catecholamine discharge due to trauma may cause ischemia in the deep capillary plexus.^13,14^ Our patient’s left eye exhibited the clinical and OCT findings of AMN in the absence of any signs of Purtscher retinopathy. Perhaps AMN and Purtscher retinopathy can be regarded as two points on a single disease spectrum that occur when the same ischemic process affects different levels.

In this article, we present a patient who developed AMN due to Purtscher retinopathy together with OCTA findings for the first time. Although there are no OCTA images from the patient’s initial presentation, his 6-month follow-up images show that the choriocapillaris was affected along with the superficial and deep capillary plexuses. The outer retinal defects observed on OCT in the early period had partially resolved and at 6 months appeared as localized disruptions in the OS/RPE line and thinning of the inner nuclear layer.

In conclusion, AMN is a clinical condition that should be kept in mind when a patient presents with post-traumatic vision loss. Although clinical diagnosis is difficult, it is easier to diagnose based on characteristic findings on OCT and OCTA.
